# Preface

**Published:** 1799-03

**Authors:** 


### PREFACE.

IN forming the plan of a *Medical and Phyſical Journal*, two eſſential objects have naturally engaged the attention, and directed the efforts of its conductors.

The firſt and preliminary conſideration, was that of rendering it a reſpectable vehicle for thoſe diſcoveries, improvements, and medical caſes, which either required to be ſpeedily laid before the Public, or which, not being of ſufficient importance to be publiſhed ſeparately, might, without ſuch an opportunity, be conſigned to oblivion.

The ſecond, and a motive no leſs cogent, was that of collecting and condenſing thoſe hints and improvements which are conſtantly iſſuing from the preſſes of Europe and America; and which, being ſcattered in voluminous and expenſive works, would be loſt to the greater number of medical practitioners.

Whether theſe two objects, ſo eſſentially involving the merits, as well as the ſucceſs of the preſent undertaking, have in ſufficient degree been attained, we ſubmit to the deciſion of the candid and unprejudiced reader.

Liberally ſupported by numerous and reſpectable correſpondents, the conductors believe that their publication has had the merit of exciting the attention of the ſtudent, and a general ſpirit of inveſtigation, among medical and phyſical enquirers, and at the ſame time it has been the means of diffuſing no little variety of inſtructive and valuable information. And as it is the primary object of a periodical work, that it ſhould become a centre of communication, the conductors congratulate themſelves on the very flattering and unexampled teſtimonies of favour they have received, by the regular and abundant ſupply of original articles, equally inſtructive and valuable.

Improvements in ſcience are naturally progreſſive: and in medicine, ſurgery, and pharmacy, this progreſs muſt be ſlow, on account of the caution with which new experiments are received, and of the great difficulty of diſtinguiſhing between fact and preconceived opinion.

### PREFACE.

The prefent volume will be found to contain, among other important articles, an accurate and comprehenfive account of one of the moft interefting phyfiological facts recorded in the hiftory of medicine, viz. the perpetual means of fuperfeding one difeafe by the introduction of another, and the difcovery of the origin of contagious difeafes, evinced in the *Inoculation of the Cow-pox.*

The botanical and chemical articles are numerous and valuable; but we fhall particularly mention only the elaborate and curious effay on the *Chemical Analyfis of Vegetables*, from the able pen of Dr. HERMBSTAEDT, of Berlin.

In the departments of anatomy and furgery, we have not hitherto been fo fortunate as in the other branches of medical fcience; yet we are not without the hope of fupplying this deficiency in our future numbers. The hiftory of anatomy, perhaps the moft effential, if not the moft neceffary part of medical ftudy, we fhall endeavour to illuftrate from the claffical papers of Profeffor SPRENGEL, of Halle; and from the difcoveries lately made by SOEMMERING, MECKEL, WALTER, and LODER, four of the moft ingenious foreign anatomifts of the prefent period.

In the *practical* department of pharmacy, we, without hefitation, promife our readers a rich harveft, as we are already in poffeffion of a confiderable quantity of new and valuable facts, tending to improve that effential branch of medical practice.

In a word, the conductors are fanctioned in the belief, that the execution of the work has not fallen fhort of their profeffions, and of the expectations excited, by the extraordinary extent of circulation which it has obtained—a circulation far greater than that of any former medical work publifhed in this country.

*London, June* 29, 1794.

---

*N. B. That the third volume may begin with the commencement of the year* 1800, *it has been judged expedient to limit the two firft volumes to five numbers each.*

